# Skin-Protective Activities of *Dioscorea batatas* Decne Peel Extracts with Differential Phenanthrene Contents

**DOI:** 10.3390/antiox15060733

**Published:** 2026-06-09

**Authors:** Yu Hyeon Kim, Taewon Kim, Jiwon Kim, Thi Yen Nhi Tran, Dongyup Hahn, Nam Joo Kang

**Affiliations:** 1 School of Food Science and Biotechnology, Kyungpook National University, Daegu 41566, Republic of Korea; uhyun095@knu.ac.kr (Y.H.K.); kjw07478@knu.ac.kr (J.K.); dohahn@knu.ac.kr (D.H.); 2 Department of Biotechnology, The Catholic University of Korea, Bucheon 14662, Republic of Korea; estomd15@catholic.ac.kr; 3 Department of Bioconvergence, Kyungpook National University, Daegu 41566, Republic of Korea; tranthiyennhi@knu.ac.kr; 4 Faculty of Chemistry, University of Science, Ho Chi Minh City 70000, Vietnam; 5 Vietnam National University, Ho Chi Minh City 70000, Vietnam; 6 Department of Integrative Biotechnology, Kyungpook National University, Daegu 41566, Republic of Korea

**Keywords:** *Dioscorea batatas* Decne peel, antioxidant, anti-melanogenesis, skin-whitening, anti-photoaging

## Abstract

**Background/Objectives**: *Dioscorea batatas* Decne (yam), which contains various bioactive compounds, has been utilized in the cosmetics industry, while most of the peel of *D. batatas* (DBP) is discarded without further use. Recent studies have shown that DBP contains higher levels of bioactive substances than the rhizome flesh. The aim of this study was to evaluate the skin biological activities of DBP extracts obtained using 70% ethanol (70% EtOH DBP), 95% ethanol (95% EtOH DBP), and ethyl acetate (EA DBP), with particular attention to their antioxidant-associated protective effects. **Methods**: Skin-related bioactivities of DBP extracts prepared using ultrasonic extraction were evaluated using in vitro tyrosinase and matrix metalloproteinase-1 (MMP-1) assays, alpha-melanocyte-stimulating hormone (α-MSH)-induced melanogenesis in B16F10 cells, ultraviolet B (UVB)-irradiated HaCaT viability assays, and Western blot analysis of pro-collagen type I alpha 1(Pro-COL1A1) and MMP-1 in HDF cells. In addition, the ABTS and DPPH radical scavenging activities of DBP extracts and representative DBP derivatives were assessed. **Results**: DBP extracts inhibited tyrosinase activity in vitro and reduced melanogenesis in B16F10 cells. DBP extracts also protected skin cells from UVB by increasing the viability of UVB-irradiated HaCaT cells. In UVB-irradiated HDF cells, DBP extracts restored Pro-COL1A1 expression and suppressed MMP-1 levels. Additionally, DBP extracts inhibited MMP-1 activity in a concentration-dependent manner. The DBP extracts themselves exhibited ABTS and DPPH radical scavenging activities, with EA DBP showing the highest vitamin C equivalent antioxidant capacity among the tested extracts. Representative DBP-derived phenanthrene compounds also showed radical scavenging activities, supporting the antioxidant potential of peel-derived phytochemicals. **Conclusions**: These findings indicate that DBP extracts possess skin-whitening and anti-photoaging effects and suggest that these protective activities may be associated with the antioxidant potential of both DBP extracts and DBP derivatives.

## 1. Introduction

The skin is the largest organ of the human body and serves as a primary barrier against physical, chemical, and environmental stressors [1,2]. In addition to its protective role, the skin is also of major esthetic importance. Structurally, the skin is composed of the epidermis, dermis, and subcutaneous fat layer [3]. The epidermis is mainly composed of keratinocytes [4]. Melanocytes are dispersed throughout the basal layer of the epidermis and produce melanin, one of the main factors that determine skin color [5]. The dermis contains collagen fibers, elastic fibers, and extracellular matrix components that are essential for maintaining skin strength and elasticity [6]. With aging, the organization and function of these structural components progressively decline, leading to reduced tissue resilience and impaired skin repair [7].

Among the external factors that accelerate skin aging, ultraviolet B (UVB) irradiation is one of the most important contributors to photoaging [8]. UVB exposure induces oxidative stress through excessive generation of reactive oxygen species (ROS), which damage cellular macromolecules and activate signaling pathways involved in inflammation, pigmentation, and extracellular matrix degradation [8,9]. In particular, UVB-induced oxidative stress activates transcription factors such as activator protein-1 (AP-1) and nuclear factor kappa-light-chain-enhancer of activated B cells (NF-κB) [10,11,12], which promote the expression of matrix metalloproteinase-1 (MMP-1), a major interstitial collagenase responsible for degradation of type I collagen in human dermal fibroblasts [12,13,14]. Increased MMP-1 activity and reduced collagen synthesis are closely associated with wrinkle formation and loss of skin elasticity [11,13,15]. Therefore, natural compounds with antioxidant potential are of considerable interest as candidates for preventing UVB-associated skin damage and photoaging [9,16].

*Dioscorea batatas* Decne (yam) is a perennial plant widely consumed in East Asia and traditionally used as both a food and medicinal resource [17,18,19]. Previous studies have reported that *D. batatas* possesses diverse biological activities, including antioxidant [20], antidiabetic [21], and anti-inflammatory effects [22]. During postharvest processing, the rhizomes of *D. batatas* are peeled, and a substantial amount of peel is discarded as waste [23]. However, recent studies have shown that *D. batatas* peel (DBP) contains higher levels of bioactive compounds, including allantoin [24], diosgenin [25], and phenanthrenes (PHE) [23], than the flesh. In addition, DBP has been reported to exhibit strong antioxidant activity [23,26], suggesting that this underutilized by-product may serve as a valuable source of functional phytochemicals. The relatively high abundance of these bioactive constituents provides a rationale for the potentially superior biological activity of DBP compared with the edible flesh. Nevertheless, comparative studies on DBP extracts prepared using different solvent systems are still limited, and the relationship between PHE enrichment and skin-related bioactivities has not been clearly established. Therefore, further investigation is warranted to better understand the skin-protective potential of DBP, particularly its anti-melanogenic and anti-photoaging activities in relation to antioxidant-associated phytochemicals.

In the present study, we investigated skin-protective effects of DBP extracts prepared using 70% or 95% ethanol, and ethyl acetate. We evaluated their anti-melanogenic and anti-photoaging activities using in vitro and cell-based models and further examined the radical scavenging activities of DBP extracts and representative DBP derivatives. In addition, PHE contents in the DBP extracts were quantitatively analyzed to explore the possible association between PHE enrichment and skin-protective bioactivity. We aimed to assess whether DBP, a food processing by-product enriched in antioxidant-associated phytochemicals, may serve as a promising natural resource for skin protection and cosmetic applications.

## 2. Materials and Methods

### 2.1. Plant Materials

*D. batatas* was purchased from Taesan-nongjang (Andong, Republic of Korea). The Chinese yam tubers were peeled off, and then the flesh and the peel were separated. The peel was washed with water and cut into slices and dried using a hot-air dryer at 65 °C.

### 2.2. Preparation of Extracts, and Isolation of Compounds

**Figure 1:**
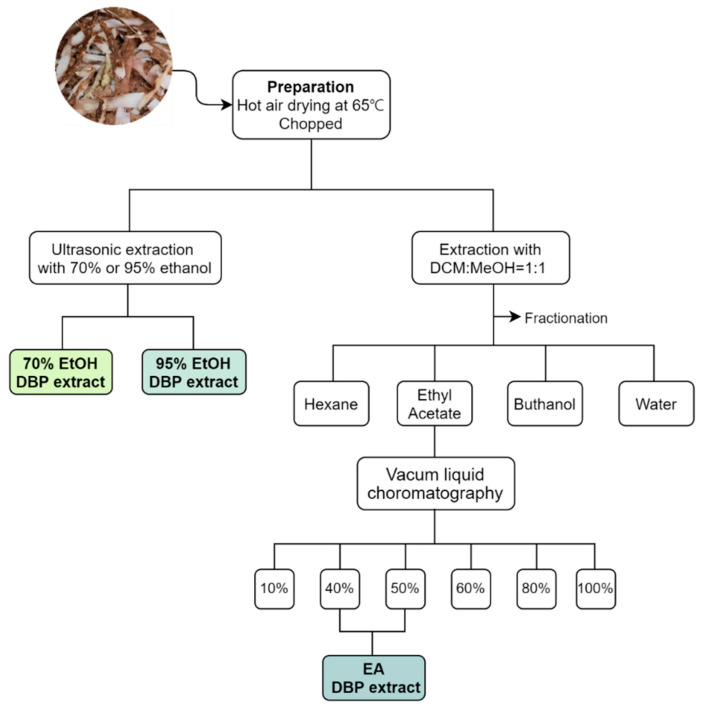
Schematic diagram of workflow for extraction of *Dioscorea batatas* Decne peel (DBP).

The dried peel of *D. batatas* was extracted with 70% ethanol (70% EtOH DBP) and 95% ethanol (95% EtOH DBP) at a solid-to-liquid ratio of 1:10 (*w*/*v*) using an ultrasonic bath (Branson 5510, Branson Ultrasonics, Danbury, CT, USA) at 25 °C for 60 min. The operating frequency was set to 40 kHz with an ultrasonic power of 135 W. The extraction yields for 70% EtOH DBP and 95% EtOH DBP were 2.11% (*w*/*w*) and 0.43% (*w*/*w*), respectively. To isolate ethyl acetate fraction (EA DBP), DBP was extracted with DCM/MeOH (Dichloromethane/methanol; 1:1, *v*/*v*) at a solid-to-liquid ratio of 1:10 (*w*/*v*) and partitioned into n-hexane, ethyl acetate, butanol, and water layers. The ethyl acetate layer was further fractionated by vacuum liquid chromatography on silica gel using an n-hexane/ethyl acetate gradient system (stepwise from 10% to 100% status of ethyl acetate), yielding six fractions. Based on the hypothesis that PHE derivatives are the major bioactive constituents, the 40% and 50% fractions showing enrichment of PHE 1, 2, and 3 were pooled to obtain the final EA DBP fraction (yield: 0.07% *w*/*w*) [23]. A simplified extraction workflow of DBP extracts is shown in Figure 1. The three compounds (PHE 1, PHE 2 and PHE 3) were isolated from *D. batatas* following the method described in the previous report [23]. Following the extraction and fractionation procedures, all organic solvents were completely evaporated under reduced pressure using a rotary evaporator (N-1000, EYELA, Tokyo, Japan). The resulting residues were lyophilized to obtain solid powdered extracts. Prior to the biological assays, these dried extracts were dissolved in DMSO.

### 2.3. Chemicals and Reagents

All samples were dissolved in dimethyl sulfoxide (DMSO; Sigma-Aldrich, St. Louis, MO, USA). Primary antibody against human MMP-1 (ab38929) was purchased from Abcam (Cambridge, UK), and Pro-collagen type I alpha 1 (Pro-COL1A1; sc-8782) was purchased from Santa Cruz Biotechnology (Dallas, TX, USA). Anti-β-actin, fetal bovine serum (FBS), and thiazolyl blue tetrazolium bromide (MTT) were purchased from Sigma-Aldrich. Dulbecco’s modified Eagle’s media (DMEM), trypsin-ethylenediaminetetraacetic acid (EDTA), and penicillin/streptomycin (P/S) were purchased from Gibco (Grand Island, NY, USA). Fibroblast basal media (FBM) and fibroblast growth kit were obtained from American Type Culture Collection (ATCC; Manassas, VA, USA). All the other reagents not mentioned were purchased from Sigma-Aldrich.

### 2.4. Cell Culture

Murine melanoma cell (B16F10 cells, ATCC^®^CRL-6475TM) and immortalized human keratinocytes (HaCaT cells) were cultured in DMEM containing 10% FBS and 1% P/S. Primary human dermal fibroblasts (HDF; ATCC^®^PCS-201-012TM) was cultured in FBM containing fibroblast growth kit-low serum and 1% P/S. All cells were incubated in 5% CO_2_ incubator at 37 °C.

### 2.5. Cell Viability Assay

The viability of B16F10, HaCaT, and HDF cells was determined by the MTT assay. The cells were seeded into 96-well plates and incubated for 24 h. Then, cells were treated with medium containing each sample at the indicated concentrations. B16F10 cells were incubated for 96 h, and HaCaT and HDF cells were incubated for 24 h. After treatment, 20 µL of 1 mg/mL MTT solution was added to each well, followed by incubation for 2 h. The medium was then replaced with 200 µL of DMSO to dissolve the formazan crystals for 30 min. The absorbance was measured at 570 nm using microplate reader (SUNRISE-Basic, Tecan, Männedorf, Switzerland).

### 2.6. Tyrosinase Activity Assay

DBP extracts were tested for mushroom tyrosinase inhibitory activity as described in the previous studies, with slight modification [27,28]. In 96-well plate, the reaction mixture consisting of 110 µL of 0.1 M potassium phosphate, 10 µL of mushroom tyrosinase sample (2000 U/mL), and 10 µL DBP extracts at the indicated concentration was shaken for 10 min. Then, 20 µL of 3 mM L-tyrosine was added and incubated for 3–5 min. The amount of dihydroxyphenylalanine (DOPA) was measured by a microplate reader at absorbance 490 nm (SUNRISE-Basic, Tecan).

### 2.7. Melanin Content Assay

Melanin content assay was performed according to the previous study with minor modification [29]. B16F10 cells were seeded at 5 × 10^4^ cells/mL into 6-well plate at and incubated for 24 h. The cells were pretreated with samples at the indicated concentrations. After 1 h, 100 nM of alpha-melanocyte stimulating hormone (α-MSH) was treated and incubated for 96 h. For measurement of extracellular melanin content in B16F10, 200 µL of cell culture medium was transferred in 96-well plate and absorbance was determined at 470 nm using a microplate reader (SUNRISE-Basic, Tecan).

### 2.8. UVB Irradiation

To investigate the protective effect of DBP extracts against UVB irradiation, HaCaT cells were seeded into 96-well plates and were cultured until they reached 80–90% confluence. After pretreatment with the samples for 1 h, the cells were rinsed twice with phosphate-buffered saline (PBS). The cells were covered with 100 µL mixture of PBS and each sample. And then, UVB irradiation was carried out by Bio-Link Crosslinker (Vilber Lourmat, Collégien, France), which has peak emits at 312 nm. Medium with each sample were added into 96-well plate and MTT assay was performed as described above. For Western blot analysis, the HDF cells were cultured until cell confluency of 80–90% and starved for 12 h in serum-free FBM. Samples were pretreated at the indicated concentration. Before UVB irradiation, HDF cells were rinsed twice and covered with a thin layer of PBS. Cells were irradiated with UVB at a dose of 20 mJ/cm^2^. After irradiation, fresh medium containing each sample was added, and the cells were further incubated for 12 h. The UVB irradiation doses were selected based on preliminary optimization and previous skin photodamage studies [30,31]. HaCaT cells were exposed to 30 mJ/cm^2^ UVB to induce moderate cytotoxicity suitable for evaluating photoprotective effects. HDF cells were exposed to 20 mJ/cm^2^ UVB to induce photoaging-related molecular responses, including altered Pro-COL1A1 and MMP-1 expression, while minimizing excessive cell damage that could interfere with Western blot analysis.

### 2.9. Western Blot Analysis

HDF cells were cultured at the density of 8 × 10^4^ cells/mL in a 60 mm TC-treated culture dish for 24 h. The cells were starved with serum-free medium for 12 h and samples were treated at the indicated concentration. Subsequently, UVB irradiation was performed as described above. Cell lysates were prepared in cell lysis buffer and total protein was quantified by DC protein assay (Bio-Rad Laboratories, Hercules, CA, USA) according to the manufacturer’s instructions. Appropriate amounts of protein were separated by 8% sodium dodecyl sulfate–polyacrylamide gel electrophoresis (SDS-PAGE) and electrotransferred to polyvinylidene fluoride membrane. After blocking in 5% skim milk, membranes were probed with primary antibody at 4 °C overnight. Then, membranes were incubated with horseradish peroxidase-conjugated secondary antibodies. The band of interest was visualized with ECL Prime Western blotting Detection Reagents (Cytiva, Marlborough, MA, USA). The band intensity was calculated using ImageJ software (version 1.46r, National Institutes of Health, Bethesda, MD, USA).

### 2.10. MMP-1 Activity Assay

The inhibition rate of MMP-1 activity was evaluated using the MMP-1 Fluorometric Drug Discovery Kit (Enzo Life Sciences, Farmingdale, NY, USA) according to the manufacturer’s instructions. In a 96-well plate, the MMP-1 catalytic domain and sample mixture was reacted at 37 °C. After about 30 min, TQ3-GABA-Pro-Cha-Abu-Smc-His-Ala-Dab(6-TAMRA)-Ala-Lys-NH2 (TQ3, quencher; GABA, 4-aminobutyric acid; Cha, L-cyclohexylalanine; Abu, 2-aminobutyricacid; Smc, S-methyl-L-cysteine; Dab, 2,4-diaminobutyric acid; 6-TAMRA, 6-tetramethylrhodamine) was treated as a substrate, and fluorescence values were measured in the excitation/emission wavelength range of 540/590 nm. The MMP-1 activity was calculated as a percentage compared to the untreated control group.

### 2.11. Radical Scavenging Activity Assay

The radical scavenging activities of DPB extracts and representative DBP-derived compounds were evaluated using 2,2′-Azino-bis(3-ethylbenzothiazoline-6-sulfonic acid) (ABTS) and 2,2-diphenyl-1-picrylhydrazyl (DPPH) assays with minor modifications of previously reported methods [32,33]. For the ABTS assay, ABTS was dissolved in methanol to a final concentration of 7 mM and reacted with 2.45 mM potassium persulfate in the dark for 16 h at room temperature. The resulting ABTS radical solution was diluted with ethanol to an absorbance of 0.70 ± 0.02 at 734 nm. Thereafter, 90 µL of the diluted ABTS solution was mixed with 10 µL of each sample in a 96-well plate, and the absorbance was measured at 734 nm after incubation for 5 min in the dark. For the DPPH assay, DPPH was dissolved in 80% methanol to a final concentration of 0.4 mg/mL and mixed with the samples at the indicated concentrations. After incubation for 30 min in the dark, the absorbance was measured at 517 nm using a microplate reader. Radical scavenging activity was expressed as vitamin C equivalent antioxidant capacity (VCEAC). Vitamin C was used as the reference antioxidant to calculate VCEAC. A calibration curve was generated using vitamin C standards at 0, 0.5, 1, 2, 4, and 8 µg/mL, with excellent linearity (*R*^2^ = 0.999). VCEAC values were expressed as µg/mL vitamin C equivalents. VCEAC was defined as the concentration of vitamin C (µg/mL) that produced radical scavenging activity equivalent to that of each tested sample under the same assay conditions. Thus, the VCEAC values indicate vitamin C-equivalent antioxidant capacity, not the concentration of the tested sample.

### 2.12. Statistical Analysis

All data are presented as mean ± standard deviation (SD) from at least duplicate measurements. Statistical significance was analyzed using Welch’s *t*-test for comparisons between each treatment group and the corresponding control group. For comparisons of radical scavenging activity among DBP extracts at different concentrations, two-way analysis of variance (ANOVA) followed by Holm–Sidak’s multiple comparisons test was used. A *p*-value of less than 0.05 was considered statistically significant.

### 2.13. Quantitative Analysis of Phenanthrenes Using HPLC

Quantitative analysis of phenanthrenes (PHE 1, PHE 2 and PHE 3) was performed using high-performance liquid chromatography (HPLC), following the method previously reported [34].

## 3. Results

### 3.1. Effect of DBP Extracts on Cell Viability

**Figure 2:**
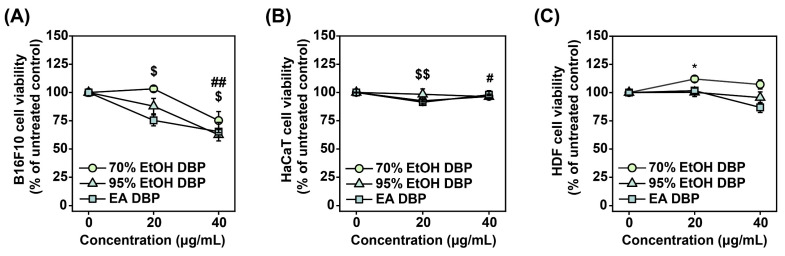
Cytotoxicity of DBP extracts in different types of cells. (**A**) B16F10 cells; (**B**) HaCaT cells; and (**C**) HDF cells. Cell viability was measured using the MTT assay at the indicated concentrations. B16F10 cells were treated with DBP extracts for 96 h, whereas HaCaT and HDF cells were treated for 24 h. Data are presented as mean ± SD (*n* = 3) and expressed as a percentage of the untreated control. Statistical significance was analyzed using Welch’s *t*-test compared with the untreated control. * *p* < 0.05 vs. untreated control of 70% EtOH DBP group; ^#^ *p* < 0.05, ^##^ *p* < 0.01 vs. untreated control of 95% EtOH DBP group; and ^$^ *p* < 0.05, ^$$^ *p* < 0.01, vs. untreated control of EA DBP group. EtOH DBP, ethanol extract of DBP; EA DBP, ethyl acetate extract of DBP.

To evaluate the cytotoxicity of DBP extracts, cell viability was assessed in B16F10, HaCaT, and HDF cells following treatment with 70% EtOH–, 95% EtOH–, and EA DBP extracts using the MTT assay. B16F10 cells were treated for 96 h to match the α-MSH-induced melanogenesis assay, whereas HaCaT and HDF cells were treated for 24 h to determine non-cytotoxic concentrations for subsequent UVB-related experiments. Because the primary objective of the cytotoxicity assay was to establish non-cytotoxic concentrations for subsequent functional assays, treatment durations were matched to the corresponding experimental protocols rather than standardized across cell lines. In B16F10 cells, all three DBP extracts reduced cell viability in a dose-dependent manner after 96 h of treatment relative to untreated control (Figure 2A). In contrast, DBP extracts treatments up to 40 μg/mL did not affect the viability of HaCaT cells and HDF cells for 24 h (Figure 2B,C). Thus, non-cytotoxic concentrations were selected for subsequent experiments. B16F10 cells were treated with ethanolic DBP extracts at concentrations up to 20 μg/mL and with EA DBP extract at 5 μg/mL. In contrast, HaCaT and HDF cells were treated with DBP extracts at concentrations up to 40 μg/mL because no significant reduction in cell viability was observed under these conditions.

### 3.2. Effect of DBP Extracts on Melanogenesis

**Figure 3:**
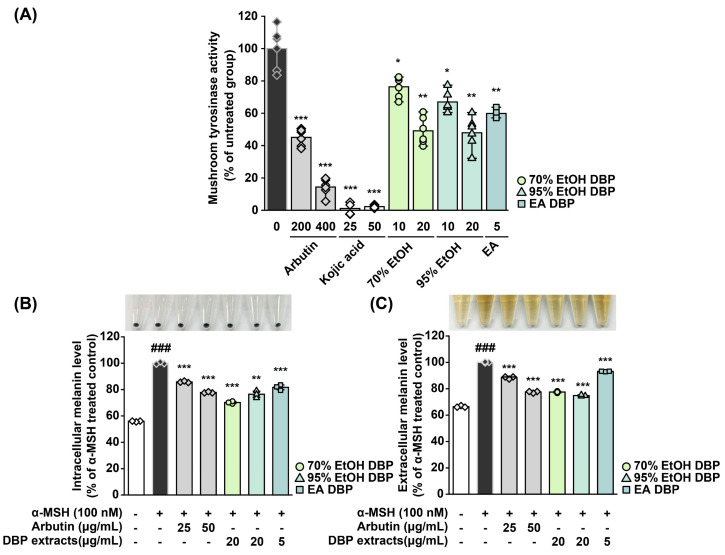
Anti-melanogenic effects of DBP extracts. (**A**) Mushroom tyrosinase activity was measured *in vitro* in the presence of DBP extracts at the indicated concentrations. Arbutin and kojic acid were used as positive controls. The black bar represents the untreated control, gray bars represent arbutin-treated groups, and white bars represent kojic acid-treated groups. Data are presented as mean ± SD (n ≥ 3). Statistical significance was analyzed using Welch’s *t*-test. * *p* < 0.05, ** *p* < 0.01, *** *p* < 0.001 vs. untreated control. (**B**,**C**) The melanin content levels of intracellular (**B**) and extracellular (**C**) were measured in B16F10 cells. Cells were pre-treated with DBP extracts for 1 h prior to α-MSH treatment and incubated for 96 h. Arbutin was used as a positive control. The white bars represent the untreated control, black bars represent the α-MSH-treated control, and gray bars represent the arbutin-treated positive control groups. Data are presented as mean ± SD (*n* = 3). Statistical significance was analyzed using Welch’s *t*-test. ** *p* < 0.01, *** *p* < 0.001 vs. α-MSH treated group; ^###^ *p* < 0.001 vs. untreated control. α-MSH, alpha-melanocyte stimulating hormone.

The mushroom tyrosinase activity of DBP extracts was evaluated in vitro (Figure 3A). The result showed that 70% EtOH DBP inhibited mushroom tyrosinase activity by 21%, 51% at 10 and 20 µg/mL, respectively. 95% EtOH DBP inhibited mushroom tyrosinase activity by 32% and 56% at 10 and 20 µg/mL compared to the untreated group, respectively. EA DBP inhibited mushroom tyrosinase activity by 41% at 5 µg/mL. These findings indicate that DBP extracts inhibit mushroom tyrosinase activity in vitro.

To further examine their anti-melanogenic effects in cells, melanin content was measured in B16F10 cells (Figure 3B,C). The results showed that DBP extracts reduced both extracellular and intracellular melanin content in each concentration. 70% EtOH DBP and 95% EtOH DBP extracts suppressed α-MSH induced extracellular melanin production by 23% and 25% at 20 μg/mL. The observed anti-melanogenic effect was comparable to or greater than that of the positive control, arbutin, at 25 and 50 μg/mL. Taken together, these results suggest that DBP extracts suppress melanogenesis in B16F10 cells, which is consistent with their inhibitory effects on tyrosinase activity.

### 3.3. Protective Effect of DBP Extracts Against UVB-Irradiated Damage

**Figure 4:**
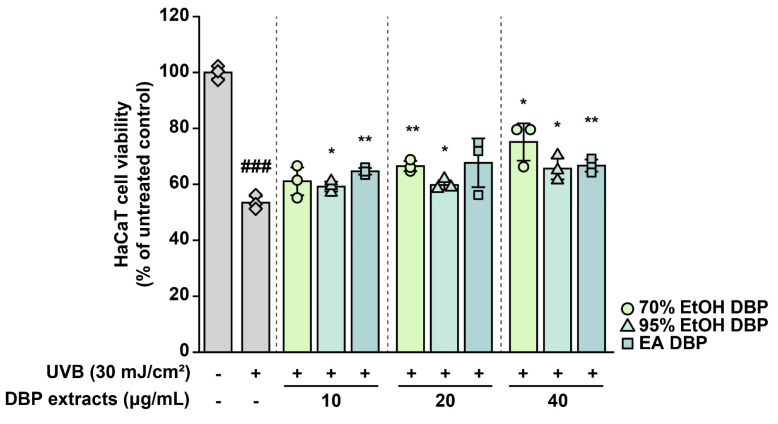
Protective effect of DBP extracts on UVB-irradiated HaCaT cells. Cell viability was measured using the MTT assay after UVB irradiation. HaCaT cells were pretreated with DBP extracts at the indicated concentrations, exposed to UVB (30 mJ/cm^2^), and incubated for 24 h. Gray bars represent the unirradiated control (left bar) and UVB-irradiated control (right bar) groups. Data are presented as mean ± SD (*n* = 3). Statistical significance was analyzed using Welch’s *t*-test. * *p* < 0.05, ** *p* < 0.01 vs. UVB-irradiated group; ^###^ *p* < 0.001 vs. unirradiated control.

To demonstrate that DBP extracts could protect HaCaT cells from UVB, MTT assay was performed after irradiation 24 h later. The cell viability was reduced by half (53% compared to control group) after UVB irradiation and moderately reversed by DBP extracts (Figure 4). The co-treatment of UVB with 70% EtOH DBP, 95% EtOH DBP, and EA DBP, respectively, showed 75%, 66%, and 67% of viability at the 40 µg/mL. This result suggests that DBP extract could attenuate UVB-induced cytotoxicity in HaCaT cells.

### 3.4. Effect of DBP Extracts on UVB-Induced Photoaging

**Figure 5:**
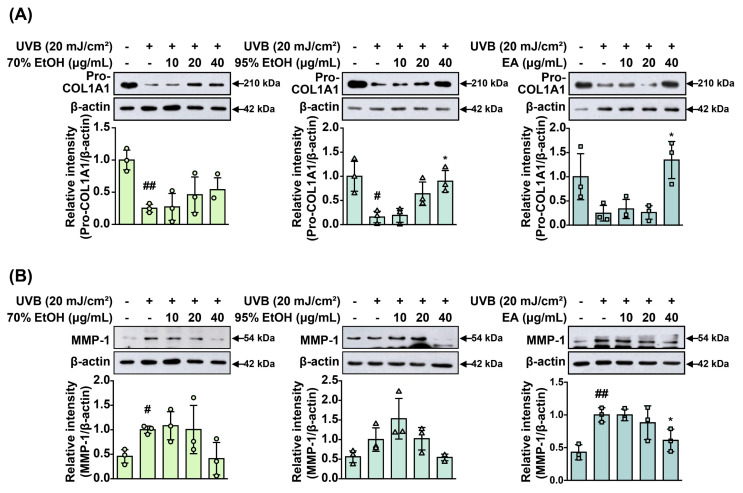
Effect of DBP extracts on Pro-COL1A1 and MMP-1 expressions in UVB-irradiated HDF cells. Expression levels of Pro-COL1A1 (**A**) and MMP-1 (**B**) were determined by Western blot analysis. HDF cells were pretreated with DBP extracts at 10, 20, 40 µg/mL for 1 h. The cells were exposed to UVB and incubated for 12 h. Protein bands were quantified relative to β-actin. The light green bars (left panels) represent the 70% EtOH DBP-treated groups, the pale green-blue bars (middle panels) represent the 95% EtOH DBP-treated groups, and the blue-green bars (right panels) represent the EA DBP-treated groups. Data are presented as mean ± SD (*n* = 3). Statistical significance was analyzed using Welch’s *t*-test. * *p* < 0.05 vs. UVB-irradiated group; ^#^ *p* < 0.05, ^##^ *p* < 0.01 vs. untreated control.

It has been reported that UVB irradiation decreases procollagen expression level, while increasing MMP-1 expression level in HDF cells. To investigate whether DBP extracts regulate skin photoaging, we measured Pro-COL1A1 and MMP-1 expression levels in HDF cells after UVB irradiation at 10, 20, and 40 µg/mL. Western blot analysis showed that decreased Pro-COL1A1 expression was restored by DBP extracts in UVB-irradiated HDF cells (Figure 5A). DBP extracts significantly restored Pro-COL1A1 expression at 40 µg/mL. In contrast, MMP-1 expression was elevated following UVB irradiation (Figure 5B). This increase was reduced by treatment with DBP extracts, with significant suppression observed at 40 µg/mL, particularly in the 95% EtOH DBP and EA DBP groups. These findings suggest that DBP extracts exert protective effects against UVB-induced photoaging in HDF cells by restoring Pro-COL1A1 expression and suppressing MMP-1 expression.

### 3.5. Inhibitory Effect of DBP Extract on MMP-1 Activity

**Figure 6:**
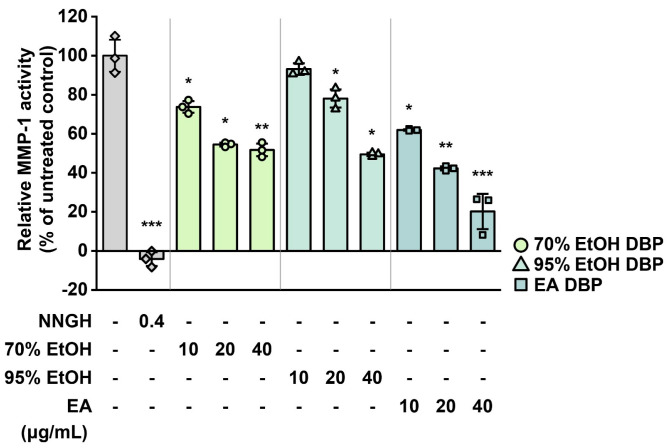
Inhibitory effects of DBP extracts on MMP-1 activity. MMP-1 activity was measured using the MMP-1 Fluorometric Drug Discovery Kit according to the manufacturer’s instructions. A reaction mixture containing MMP-1 assay buffer, the MMP-1 catalytic domain, and DBP extracts at the indicated concentrations was incubated at 37 °C for 30 min. NNGH (0.4 µg/mL; 1.3 µM) was used as a positive control. Gray bars represent the untreated control (left bar) and NNGH-treated positive control (right bar). Data are presented as mean ± SD (*n* = 3). Statistical significance was analyzed using Welch’s *t*-test. * *p* < 0.05; ** *p* < 0.01; *** *p* < 0.001 vs. untreated group. NNGH, N-Isobutyl-N-(4-methoxyphenylsufonyl) glycyl hydroxamic acid.

Additionally, the MMP-1 inhibition activity of DBP extracts was measured in vitro (Figure 6). DBP extracts inhibited MMP-1 catalytic activity compared with the untreated control group. 70% EtOH DBP extract inhibited MMP-1 activity by 26.3%, 45.5% and 48.3% at 10, 20 and 40 µg/mL, respectively. 95% EtOH DBP extract inhibited MMP-1 activity by 6.9%, 21.9% and 50.6% at 10, 20 and 40 µg/mL. EA DBP extract inhibited MMP-1 activity by 38.1%, 57.7% and 79.8% at 10, 20 and 40 µg/mL. These results indicate that the DBP extracts exhibit inhibitory activity against MMP-1 in a dose-dependent manner.

### 3.6. Radical Scavenging Activity of DBP Extracts and Derivatives

**Figure 7:**
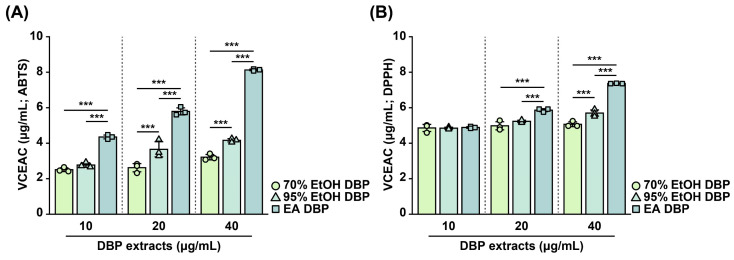
Radical scavenging activity of DBP extracts. ABTS radical scavenging activity (**A**) and DPPH radical scavenging activity (**B**) of 70% EtOH DBP, 95% EtOH DBP, and EA DBP extracts. Radical scavenging activity was expressed as VCEAC, which indicates the concentration of vitamin C showing radical scavenging activity equivalent to that of each tested sample. Data are presented as mean ± SD (*n* = 3). Statistical significance was analyzed using two-way ANOVA followed by Holm–Sidak’s multiple comparisons test for comparisons among extracts at the same concentration. *** *p* < 0.001. VCEAC, vitamin C equivalent antioxidant capacity.

To further investigate whether the skin-protective effects of DBP extracts may be associated with their antioxidant properties, the radical scavenging activities of the three DBP extracts were evaluated using ABTS and DPPH assays (Figure 7). Radical scavenging activity was expressed as vitamin C equivalent antioxidant capacity (VCEAC). In the ABTS assay, all DBP extracts showed measurable VCEAC values at the tested concentrations. Among the extracts, EA DBP exhibited the highest ABTS radical scavenging activity, followed by 95% EtOH DBP and 70% EtOH DBP. This trend was particularly evident at 40 µg/mL, where EA DBP showed the highest VCEAC value among the tested extracts. Similarly, in the DPPH assay, EA DBP showed the strongest radical scavenging activity, whereas 70% EtOH DBP and 95% EtOH DBP exhibited relatively lower but measurable VCEAC values. These results demonstrate that the DBP extracts themselves possess radical scavenging activity and support the antioxidant potential of DBP-derived preparations.

**Figure 8:**
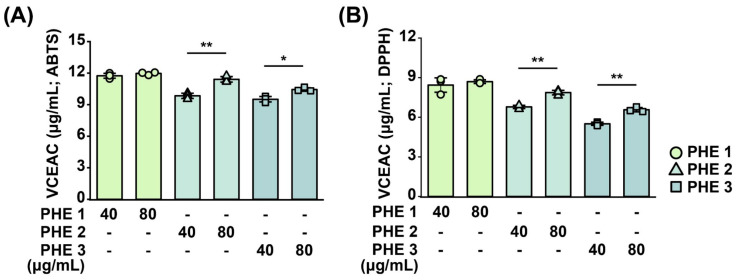
Effects of PHE derivatives on radical scavenging activity. ABTS radical scavenging activity (**A**) and DPPH radical scavenging activity (**B**) of PHE derivatives. Radical scavenging activity was expressed as VCEAC. Data are presented as mean ± SD (*n* = 3). Statistical significance between 40 and 80 µg/mL within each PHE derivative was analyzed using Welch’s *t*-test. * *p* < 0.05; ** *p* < 0.01. VCEAC, vitamin C equivalent antioxidant capacities; PHE, phenanthrene.

To further investigate whether the skin-protective effects of DBP may be associated with antioxidant constituents, the radical scavenging activities of three representative DBP derivatives (PHE 1, PHE 2, and PHE 3) were evaluated using ABTS and DPPH assays (Figure 8). The antioxidant capacity of each compound was also expressed as VCEAC. In the ABTS assay, the VCEAC values of PHE 1 were 11.75 and 11.97 µg/mL at 40 and 80 µg/mL, respectively, whereas those of PHE 2 were 9.84 and 11.41 µg/mL, and those of PHE 3 were 9.51 and 10.44 µg/mL at the same concentrations. In the DPPH assay, the VCEAC values of PHE 1 were 9.36 and 9.76 µg/mL at 40 and 80 µg/mL, respectively, whereas those of PHE 2 were 7.80 and 9.03 µg/mL, and those of PHE 3 were 6.47 and 7.62 µg/mL. These results indicate that representative DBP derivatives possess measurable radical scavenging activity, as shown by their quantifiable VCEAC values at both tested concentrations in the ABTS and DPPH assays. These findings support the antioxidant potential of DBP derivatives and suggest that they may contribute, at least in part, to the antioxidant-associated skin-protective effects of DBP. Further studies using complementary radical scavenging assays will be useful to more fully characterize their antioxidative properties.

### 3.7. Quantitative Analysis of Phenanthrenes

The quantitative analysis of bioactive PHEs was performed using the validated HPLC analysis. The linearity of the calibration curves was satisfactory, with determination coefficients (*R*^2^) of 0.9998 for PHE 1, 0.9997 for PHE 2, and 0.9973 for PHE 3 in the concentration range of 0.625–20.00 µg/mL. As shown in Table 1, PHE 1, PHE 2 and PHE 3 were found in all investigated peel extracts. In 70% EtOH DBP, the contents of PHE 1, PHE 2, and PHE 3 were 171.98 ± 3.70, 340.85 ± 1.60, and 286.93 ± 2.93 µg/g of dried extract, respectively. 95% EtOH DBP exhibited higher PHE contents, with 681.74 ± 43.35 µg/g of dried extract for PHE 1, 1300.34 ± 104.12 µg/g of dried extract for PHE 2, and 1011.43 ± 88.44 µg/g of dried extract for PHE 3. For EA DBP, the concentrations of PHE 1, PHE 2, and PHE 3 were determined to be 2752.74 ± 83.60, 5764.97 ± 96.31, and 3682.80 ± 96.05 µg/g of dried extract, respectively.

## 4. Discussion

The genus of *Dioscorea* has traditionally been used as both a food and medicinal resource in Asia [17,18,19]. Since the flesh of the rhizome of *D. batatas* Decne has various phytochemicals, its extracts and bioactive compounds have been studied intensively [26,35,36]. Even though recent studies have revealed that the peel of *D. batatas* also possesses wide range of biological activities, the role of the peel in skin health has not been researched. In this study, we demonstrated that DBP extracts exhibit dermal biological activities by using in vitro assay and cell-based assay. Thus, DBP extracts may have potential for cosmetic applications. Initially, we evaluated the cytotoxicity of DBP extracts in different types of skin cells. For melanin content analysis, B16F10 cells were treated with each DBP extract for 96 h (Figure 2A). In addition, the photoprotective effect was investigated by treating DBP extracts on HaCaT cells and HDF cells for 24 h (Figure 2B,C). Each sample was treated to a non-toxic concentration according to the results.

Melanin is the pigment that determines skin color and plays an important protective role against ultraviolet radiation [37,38]. Melanogenesis is initiated by tyrosinase, the rate-limiting enzyme that catalyzes the hydroxylation of L-tyrosine to L-DOPA and the subsequent oxidation of L-DOPA to dopaquinone [39]. Therefore, inhibition of tyrosinase activity is a widely used strategy for skin-whitening and anti-hyperpigmentation applications. In the present study, DBP extracts inhibited mushroom tyrosinase activity and reduced melanin content in α-MSH-stimulated B16F10 cells, indicating their anti-melanogenic potential. Previous studies have reported that Dioscorea flesh extracts and their bioactive constituents can modulate melanogenesis-related pathways [40,41,42]. In particular, diosgenin, a steroidal saponin abundant in Dioscorea species, has been shown to suppress melanogenesis in B16 melanoma cells through activation of the PI3K signaling pathway [43]. Taken together, these findings suggest that the anti-melanogenic activity of DBP extracts may be attributable to bioactive phytochemicals enriched in the peel. Nevertheless, because melanogenesis was evaluated using B16F10 murine melanoma cells and mushroom tyrosinase, further studies using human melanocytes and human tyrosinase-based systems are warranted to confirm the anti-melanogenic effects of DBP extracts.

We next investigated whether DBP extracts could protect skin cells against UVB-induced photoaging. UVB irradiation is a major contributor to extrinsic skin aging because it induces reactive oxygen species, disrupts cellular antioxidant defense systems, and promotes DNA damage [9,14]. Consistent with these harmful effects, UVB exposure reduced cell viability in HaCaT cells, whereas DBP extracts improved cell survival under UVB-irradiated conditions. These findings suggest that DBP extracts may exert photoprotective effects in epidermal cells.

Photoaging is also closely associated with dysregulation of extracellular matrix homeostasis. UVB activates signaling pathways involving transcription factors such as AP-1 and NF-κB, which promote the expression of matrix metalloproteinases, including MMP-1 [11]. Because MMP-1 degrades interstitial collagen and contributes to dermal structural damage, suppression of MMP-1 and preservation of procollagen are important targets in anti-photoaging research. AP-1 promotes the expression and activity of MMP-1, which degrades connective tissue such as type I collagen and indirectly diminish collagen synthesis [12]. It was established that UV irradiation induces collagen breakdown and decreases procollagen synthesis in eight hours [44]. Previous study reported that ethanol extract of *D. batatas* flesh regulates procollagen and MMP-1 expression in TNF-α-induced HDF cells [45]. Our findings extend these observations by suggesting that peel-derived extracts also possess anti-photoaging-related activity under UVB-induced conditions. Although the underlying mechanisms were not directly investigated in the present study, UVB-induced oxidative stress is known to activate MAPK signaling pathways, including ERK, JNK, and p38, which subsequently regulate AP-1 and NF-κB activity. These transcription factors play critical roles in the induction of MMP-1 expression and collagen degradation during photoaging. Therefore, the observed suppression of MMP-1 and restoration of Pro-COL1A1 expression by DBP extracts may be associated with modulation of MAPK/AP-1- and/or NF-κB-mediated signaling pathways. Further mechanistic studies are needed to verify this possibility.

Among the tested extracts, EA DBP generally showed stronger anti-photoaging-related activity than the ethanolic extracts. The enhanced anti-photoaging and anti-melanogenic efficacy of EA DBP is closely correlated with the selective enrichment of these bioactive PHEs. According to our quantitative analysis (Table 1), the concentrations of PHE 1, 2, and 3 in the EA fraction were notably higher than those in the 70% and 95% ethanolic extracts. In the present study, individual PHEs demonstrated significant ABTS and DPPH radical scavenging activities (Figure 8), supporting their roles as antioxidant agents. Since UVB-induced oxidative stress triggers upstream signaling pathways, such as AP-1 and NF-κB, which upregulate MMP-1 expression and induce cytotoxicity, the concentration of these antioxidants within the EA fraction likely provides the observed cellular protection and enzymatic inhibition. Consequently, the enrichment of PHEs via ethyl acetate fractionation accounts for the enhanced functional performance of EA DBP. This difference may be attributable to variations in phytochemical composition resulting from the extraction and fractionation procedures [46]. The polarity between solvent and compound is one of the main factors that affect the isolation effectiveness [47]. It is also known that extraction with a mixed solvent rather than a single solvent is more suitable for isolation of phenolic compounds from plants [48]. As shown in Figure 1, 70% EtOH and 95% EtOH used for extraction are the mixtures of EtOH and water, and EA DBP is a fraction of DCM/MeOH mixed solvent extracts. The variation in biological activity might result from the difference in isolated compounds according to the extraction solvent. Furthermore, due to the harmful effect on the human body, the use of methanol in cosmetic formulations is restricted by regulatory guidelines [49,50]. Thus, optimizing extraction methods is important for precise analysis of the functional compounds of DBP.

The ABTS and DPPH assays performed using the DBP extracts provide direct evidence that the extracts themselves possess radical scavenging activity. All three DBP extracts showed measurable VCEAC values, supporting the antioxidant potential of the extracts as well as that of the representative DBP derivatives. Among the tested extracts, EA DBP showed relatively higher VCEAC values in both ABTS and DPPH assays, which is in line with its higher phenanthrene content. These findings suggest that the antioxidant-associated properties of the whole extracts may be related to their skin-protective effects, and that PHE enrichment may partly explain the enhanced activity of EA DBP. In addition to the extract-level antioxidant activity, representative DBP-derived phenanthrene compounds also exhibited ABTS and DPPH radical scavenging activities, further supporting the contribution of peel-derived phytochemicals to the antioxidant potential of DBP. Among the tested compounds, PHE 1 showed the highest radical scavenging activity, followed by PHE 2 and PHE 3. Although these chemical assays do not directly demonstrate intracellular ROS scavenging, they suggest that the skin-protective effects of DBP extracts may be associated with redox-active constituents present in the peel.

Taken together, our findings demonstrate that DBP extracts suppress melanogenesis in α-MSH-induced B16F10 cells and attenuate UVB-induced photoaging-related responses in skin cell models. These effects were associated with inhibition of tyrosinase and MMP-1 activity, restoration of Pro-COL1A1 expression, and reduction in MMP-1 expression. Among the tested extracts, EA DBP consistently exhibited stronger anti-photoaging-related activity, particularly with respect to suppression of MMP-1 activity and expression. To further investigate the phytochemical characteristics associated with these biological effects, quantitative HPLC analysis of representative PHE 1–3 was performed. To further support this association, representative PHE compounds isolated from DBP were subjected to ABTS and DPPH radical scavenging assays and were found to possess antioxidant activity. Nevertheless, because DBP extracts contain diverse phytochemical constituents, the potential contribution of synergistic interactions among multiple compounds cannot be excluded and warrants further investigation. Although further studies are needed to clarify the contribution of phenanthrene compounds and the underlying molecular mechanisms, the present results suggest that DBP extracts may serve as promising candidates for cosmetic and dermocosmetic applications.

## 5. Conclusions

DBP extracts exhibited anti-melanogenic and anti-photoaging activities by inhibiting tyrosinase activity, reducing melanin production in B16F10 cells, protecting HaCaT cells against UVB-induced cytotoxicity, restoring Pro-COL1A1 expression, and suppressing both the expression and activity of MMP-1 in HDF cells. In addition, DBP extracts themselves showed ABTS and DPPH radical scavenging activities, and representative DBP derivatives also exhibited measurable antioxidant activity. Notably, EA DBP showed both strong biological activity and the highest phenanthrene contents among the tested extracts, suggesting a possible association among PHE enrichment, antioxidant capacity, and the skin-protective effects of DBP. Collectively, these findings suggest that DBP is a promising value-added natural resource for skin-whitening and anti-photoaging applications and that its protective effects may be associated with antioxidative mechanisms. Further mechanistic studies are needed and will be pursued to define how DBP derivatives, particularly phenanthrenes, regulate MMP-1 expression and activity.

## Figures and Tables

**Table 1:** Linearity (*R*^2^) and contents of PHE 1–3 in *D. batatas* peel extracts.

*R*^2^ (Determination Coefficient)		Linear Regression Equations	Content (µg/g of Dried Extract)		
			70% EtOH DBP	95% EtOH DBP	EA DBP
PHE 1	0.9998	y = 61992x + 8058.4	171.98 ± 3.69	681.74 ± 43.35	2752.74 ± 83.60
PHE 2	0.9997	y = 40706x + 10042	340.85 ± 1.60	1300.34 ± 104.12	5764.97 ± 96.31
PHE 3	0.9973	y = 60966x + 28427	286.93 ± 2.94	1011.43 ± 88.44	3682.80 ± 96.05

Values represent the means ± SD (*n* = 3).

## Data Availability

The raw data supporting the conclusions of this article will be made available by the authors on request.

## Abbreviations

The following abbreviations are used in this manuscript:

DBP	*Dioscorea batatas* Decne peel
EtOH	Ethanol
EtOH DBP	Ethanol extracts of *Dioscorea batatas* Decne peel
EA	Ethyl acetate
EA DBP	Ethyl acetate extracts of *Dioscorea batatas* Decne peel
DCM	Dichloromethane
MeOH	Methanol
DMSO	Dimethyl sulfoxide
MTT	Thiazolyl blue tetrazolium bromide
FBS	Fetal bovine serum
DMEM	Dulbecco’s modified Eagle’s media
EDTA	Ethylenediaminetetraacetic acid
FBM	Fibroblast basal media
PBS	Phosphate-buffered saline
SDS-PAGE	Sodium dodecyl sulfate–polyacrylamide gel electrophoresis
UVB	Ultraviolet B
AP-1	Activator protein-1
NF-κB	Nuclear factor kappa-light-chain-enhancer of activated B cells
MMP-1	Matrix metalloproteinase-1
Pro-COL1A1	Pro-collagen type I alpha 1
α-MSH	Alpha-melanocyte-stimulating hormone
DOPA	Dihydroxyphenylalanine
ABTS	2,2′-Azino-bis(3-ethylbenzothiazoline-6-sulfonic acid)
DPPH	2,2-Diphenyl-1-picrylhydrazyl

## References

[1] Hadgraft J. (2001). Skin, the Final Frontier. Int. J. Pharm..

[2] Boer M., Duchnik E., Maleszka R., Marchlewicz M. (2016). Structural and Biophysical Characteristics of Human Skin in Maintaining Proper Epidermal Barrier Function. Adv. Dermatol. Allergol..

[3] Arda O., Goksugur N., Tuzun Y. (2014). Basic Histological Structure and Functions of Facial Skin. Clin. Dermatol..

[4] Brody I. (1960). The Ultrastructure of the Tonofibrils in the Keratinization Process of Normal Human Epidermis. J. Ultrastruct. Res..

[5] Lin J.Y., Fisher D.E. (2007). Melanocyte Biology and Skin Pigmentation. Nature.

[6] Woo W.-M. (2019). Skin Structure and Biology. Imaging Technologies and Transdermal Delivery in Skin Disorders.

[7] Vierkotter A., Krutmann J. (2012). Environmental Influences on Skin Aging and Ethnic-Specific Manifestations. Dermato-Endocrinology.

[8] Fisher G.J., Kang S., Varani J., Bata-Csorgo Z., Wan Y., Datta S., Voorhees J.J. (2002). Mechanisms of Photoaging and Chronological Skin Aging. Arch. Dermatol..

[9] Pandel R., Poljsak B., Godic A., Dahmane R. (2013). Skin Photoaging and the Role of Antioxidants in Its Prevention. ISRN Dermatol..

[10] Pittayapruek P., Meephansan J., Prapapan O., Komine M., Ohtsuki M. (2016). Role of Matrix Metalloproteinases in Photoaging and Photocarcinogenesis. Int. J. Mol. Sci..

[11] Fisher G.J., Wang Z., Datta S.C., Varani J., Kang S., Voorhees J. (1997). Pathophysiology of Premature Skin Aging Induced by Ultraviolet Light. N. Engl. J. Med..

[12] Fisher G.J., Voorhees J.J. (1998). Molecular Mechanisms of Photoaging and Its Prevention by Retinoic Acid: Ultraviolet Irradiation Induces MAP Kinase Signal Transduction Cascades That Induce Ap-1-Regulated Matrix Metalloproteinases That Degrade Human Skin in Vivo. J. Investig. Dermatol. Symp. Proc..

[13] Fisher G.J., Datta S.C., Talwar H.S., Wang Z.Q., Varani J., Kang S., Voorhees J.J. (1996). Molecular Basis of Sun-Induced Premature Skin Ageing and Retinoid Antagonism. Nature.

[14] Dong K.K., Damaghi N., Picart S.D., Markova N.G., Obayashi K., Okano Y., Masaki H., Grether-Beck S., Krutmann J., Smiles K. (2008). UV-Induced DNA Damage Initiates Release of MMP-1 in Human Skin. Exp. Dermatol..

[15] Chen V.L., Fleischmajer R., Schwartz E., Palaia M., Timpl R. (1986). Immunochemistry of Elastotic Material in Sun-Damaged Skin. J. Investig. Dermatol..

[16] Cavinato M., Waltenberger B., Baraldo G., Grade C.V.C., Stuppner H., Jansen-Durr P. (2017). Plant Extracts and Natural Compounds Used Against UVB-Induced Photoaging. Biogerontology.

[17] Yang M.H., Chin Y.W., Yoon K.D., Kim J. (2014). Phenolic Compounds with Pancreatic Lipase Inhibitory Activity from Korean Yam (*Dioscorea opposita*). J. Enzym. Inhib. Med. Chem..

[18] Kumar S., Das G., Shin H.S., Patra J.K. (2017). *Dioscorea* spp. (A Wild Edible Tuber): A Study on Its Ethnopharmacological Potential and Traditional Use by the Local People of Similipal Biosphere Reserve, India. Front. Pharmacol..

[19] Oh M.H., Houghton P.J., Whang W.K., Cho J.H. (2004). Screening of Korean Herbal Medicines Used to Improve Cognitive Function for Anti-Cholinesterase Activity. Phytomedicine.

[20] Hou W.C., Lee M.H., Chen H.J., Liang W.L., Han C.H., Liu Y.W., Lin Y.H. (2001). Antioxidant Activities of Dioscorin, the Storage Protein of Yam (*Dioscorea batatas* Decne) Tuber. J. Agric. Food Chem..

[21] Go H.K., Rahman M.M., Kim G.B., Na C.S., Song C.H., Kim J.S., Kim S.J., Kang H.S. (2015). Antidiabetic Effects of Yam (*Dioscorea batatas*) and Its Active Constituent, Allantoin, in a Rat Model of Streptozotocin-Induced Diabetes. Nutrients.

[22] Jin M., Suh S.J., Yang J.H., Lu Y., Kim S.J., Kwon S., Jo T.H., Kim J.W., Park Y.I., Ahn G.W. (2010). Anti-Inflammatory Activity of Bark of *Dioscorea batatas* Decne through the Inhibition of INOS and COX-2 Expressions in RAW264.7 Cells via NF-KappaB and ERK1/2 Inactivation. Food Chem. Toxicol..

[23] Kim M., Gu M.J., Lee J.-G., Chin J., Bae J.-S., Hahn D. (2019). Quantitative Analysis of Bioactive Phenanthrenes in *Dioscorea batatas* Decne Peel, a Discarded Biomass from Postharvest Processing. Antioxidants.

[24] Fu Y.C., Huang P.Y. (2006). Quantitative Analysis of Allantoin and Allantoic Acid in Yam Tuber, Mucilage, Skin and Bulbil of the *Dioscorea* Species. Food Chem..

[25] Lin J.T., Yang D.J. (2008). Determination of Steroidal Saponins in Different Organs of Yam (*Dioscorea pseudojaponica* Yamamoto). Food Chem..

[26] Hsu C.K., Yeh J.Y., Wei J.H. (2011). Protective Effects of the Crude Extracts from Yam (*Dioscorea alata*) Peel on Tert-butylhydroperoxide-Induced Oxidative Stress in Mouse Liver Cells. Food Chem..

[27] Kim J.H., Kim D.H., Cho K.M., Kim K.H., Kang N.J. (2018). Effect of 3,6-Anhydro-l-galactose on Alpha-Melanocyte Stimulating Hormone-Induced Melanogenesis in Human Melanocytes and a Skin-Equivalent Model. J. Cell Biochem..

[28] Tsuboi T., Kondoh H., Hiratsuka J., Mishima Y. (1998). Enhanced Melanogenesis Induced by Tyrosinase Gene-Transfer Increases Boron-Uptake and Killing Effect of Boron Neutron Capture Therapy for Amelanotic Melanoma. Pigment. Cell Res..

[29] Matsuda H., Nakamura S., Kubo M. (1994). Studies of Cuticle Drugs from Natural Sources. II. Inhibitory Effects of Prunus Plants on Melanin Biosynthesis. Biol. Pharm. Bull..

[30] Sun X., Kim A., Nakatani M., Shen Y., Liu L. (2016). Distinctive Molecular Responses to Ultraviolet Radiation between Keratinocytes and Melanocytes. Exp. Dermatol..

[31] Li L., Huang T., Lan C., Ding H., Yan C., Dou Y. (2019). Protective Effect of Polysaccharide from *Sophora japonica* L. Flower Buds against UVB Radiation in a Human Keratinocyte Cell Line (HaCaT Cells). J. Photochem. Photobiol. B.

[32] Re R., Pellegrini N., Proteggente A., Pannala A., Yang M., Rice-Evans C. (1999). Antioxidant Activity Applying an Improved ABTS Radical Cation Decolorization Assay. Free Radic. Biol. Med..

[33] Brand-Williams W., Cuvelier M.E., Berset C. (1995). Use of a Free Radical Method to Evaluate Antioxidant Activity. LWT Food Sci. Technol..

[34] Kim H., Cao T.Q., Yeo C.E., Shin S.H., Kim H., Hong D.H., Hahn D. (2022). Development and Validation of Quantitative Analysis Method for Phenanthrenes in Peels of the *Dioscorea* Genus. J. Microbiol. Biotechnol..

[35] Byeon S., Oh J., Lim J.S., Lee J.S., Kim J.S. (2018). Protective Effects of *Dioscorea batatas* Flesh and Peel Extracts against Ethanol-Induced Gastric Ulcer in Mice. Nutrients.

[36] Lim J.S., Oh J., Byeon S., Lee J.S., Kim J.-S. (2018). Protective Effect of *Dioscorea batatas* Peel Extract Against Intestinal Inflammation. J. Med. Food.

[37] Brenner M., Hearing V.J. (2008). The Protective Role of Melanin against UV Damage in Human Skin. Photochem. Photobiol..

[38] Costin G.E., Hearing V.J. (2007). Human Skin Pigmentation: Melanocytes Modulate Skin Color in Response to Stress. FASEB J..

[39] Hearing V.J., Jimenez M. (1987). Mammalian Tyrosinase—The Critical Regulatory Control Point in Melanocyte Pigmentation. Int. J. Biochem..

[40] Fujihara K., Takahashi K., Koyama K., Kinoshita K. (2017). Triterpenoid Saponins from *Polaskia chichipe* Backbg. and Their Inhibitory or Promotional Effects on the Melanogenesis of B16 Melanoma Cells. J. Nat. Med..

[41] Kawabata T., Cui M.Y., Hasegawa T., Takano F., Ohta T. (2011). Anti-Inflammatory and Anti-Melanogenic Steroidal Saponin Glycosides from Fenugreek (*Trigonella foenum-graecum* L.) Seeds. Planta Med..

[42] Lee S.-Y., Yoo D.-H., Joo D.-H., Lee J.-Y. (2015). Inhibitory Efficacy of Dioscoreae Rhizoma on MITF, TRP-1, TRP-2, Tyrosinase, PKA and ERK Expression in Melanoma Cells (B16F10). Korean J. Herbol..

[43] Lee J., Jung K., Kim Y.S., Park D. (2007). Diosgenin Inhibits Melanogenesis through the Activation of Phosphatidylinositol-3-Kinase Pathway (PI3K) Signaling. Life Sci..

[44] Quan T., He T., Kang S., Voorhees J.J., Fisher G.J. (2002). Ultraviolet Irradiation Alters Transforming Growth Factor Beta/Smad Pathway in Human Skin In Vivo. J. Investig. Dermatol..

[45] Kim D.S., Jeon B.K., Lim N.Y., Mun Y.J., Lee Y.E., Woo W.H. (2013). Ethanol Extract of *Dioscorea batatas* Stimulates Procollagen Production and Reduces UVB-Induced MMPs Activity in Skin. J. Physiol. Pathol. Korean Med..

[46] Luthria D.L., Biswas R., Natarajan S. (2007). Comparison of Extraction Solvents and Techniques Used for the Assay of Isoflavones from Soybean. Food Chem..

[47] Cai Y., Luo Q., Sun M., Corke H. (2004). Antioxidant Activity and Phenolic Compounds of 112 Traditional Chinese Medicinal Plants Associated with Anticancer. Life Sci..

[48] Kim K.C., Kim J.-S. (2020). Effect of Varying Ethanol Concentrations on the Extraction Properties and Physiological Activity of *Artemisia annua* L.. Korean J. Food Sci. Technol..

[49] Staff C. (1989). Final Report on the Safety Assessment of Ethyl Acetate and Butyl Acetate. J. Med. Toxicol..

[50] Kim S., Choi M., Kim D., Kim M., Park S., Min C., Choi B., Kang S., Kim E. (2013). Establishment for Methanol Analytical Method in Cosmetics. Korean Soc. Food Drug Cosmet. Regul. Sci. (KFDC).

